# Acute Negative Pressure Pulmonary Edema Following Upper Gastrointestinal (GI) Endoscopy: A Case Report

**DOI:** 10.7759/cureus.84461

**Published:** 2025-05-20

**Authors:** Abdelkader Boukharta, Khalid Bouti, Sanaa Hammi

**Affiliations:** 1 Department of Respiratory Medicine, Mohammed VI University Hospital Center, Tangier, MAR; 2 Department of Pulmonology, Mohammed VI University Hospital Center, Tangier, MAR

**Keywords:** acute pulmonary edema with negative pressure, bilateral alveolar syndrome, case report, complication, endoscopy, sedation

## Abstract

Acute pulmonary edema with negative pressure (APENP) is a rare respiratory complication caused by acute upper airway obstruction, most often occurring during the postoperative period.

We report a case of acute pulmonary edema with negative pressure outside of orotracheal intubation, occurring in a 65-year-old Moroccan woman, admitted for respiratory distress following digestive endoscopy under sedation without tracheal intubation. A chest X-ray after the procedure showed diffuse bilateral alveolar syndrome. Transthoracic echocardiography ruled out cardiac origin. Management was based on oxygen therapy with diuretics without the need for mechanical ventilation. The clinical assessment showed rapid improvement, and a second chest X-ray on the second day of treatment demonstrated a good resolution of the initial alveolar syndrome.

A good understanding of this pathological entity allows for a quick diagnosis and optimal management of this complication.

## Introduction

Acute pulmonary edema with negative pressure (APENP) is a respiratory complication due to upper airway obstruction (UAO), which most often occurs in the postoperative period under general anesthesia with orotracheal intubation. It is highly suspected in cases of hypoxemia associated with dyspnea following tracheal extubation after general anesthesia. The frequency in the anesthetic context ranges from 0.05% to 0.1%. However, this prevalence is often underestimated due to a lack of awareness of this complication [1]. Here, we report the case of APENP occurring after upper gastrointestinal (GI) endoscopy performed under sedation without intubation.

## Case presentation

A 65-year-old Moroccan woman, with a history of localized sigmoid adenocarcinoma treated surgically 11 years ago, was admitted to our facility presenting with the sudden onset of stage 4 Modified Medical Research Council (mMRC) Dyspnea Scale, associated with coughing and mucous expectoration. The symptoms occurred after a sedated digestive endoscopy performed for dysentery syndrome. The patient began to desaturate during the procedure. On clinical examination upon admission, the patient was conscious, with no signs of respiratory distress. Respiratory rate, 28 breaths per minute; SpO₂, 88% on room air, improving to 95% with 2 L of oxygen; bilateral basal crackles present.

**Figure 1:**
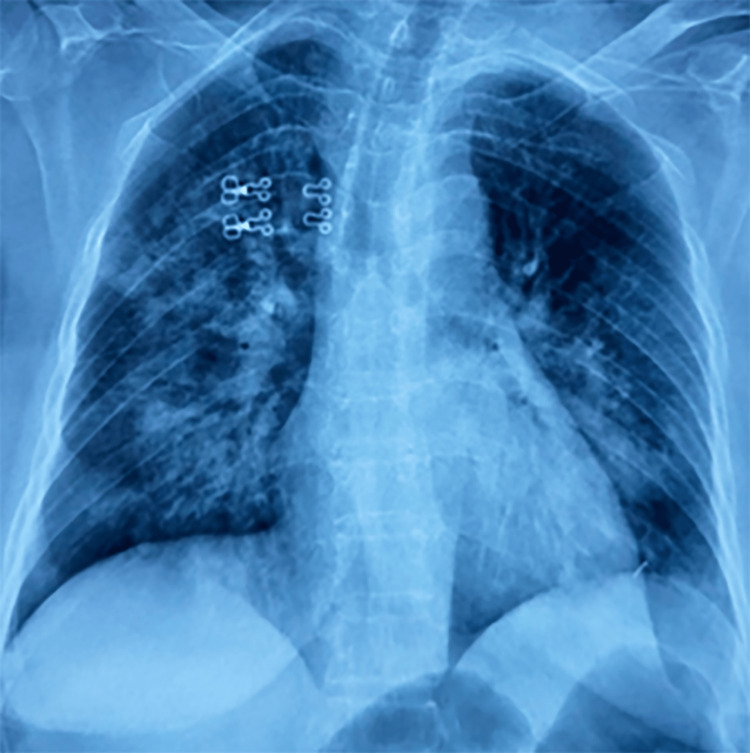
Chest X-ray (frontal view). The chest X-ray after the procedure revealed bilateral alveolar syndrome.

The chest X-ray after the procedure revealed bilateral alveolar syndrome (Figure 1).

The laboratory findings show normal hematological, renal, hepatic, and electrolyte parameters, except for mild anemia (Table 1).

**Table 1:** Laboratory findings. AST, aspartate aminotransferase; SGOT, serum glutamic-oxaloacetic transaminase; ALT, alanine aminotransferase; SGPT, serum glutamic-pyruvic transaminase

Test	Result	Units
White blood cells (WBCs)	6.63	×10³/µL
Hemoglobin (HGB)	12.8	g/dL
Hematocrit	38.2	%
Platelets (PLT)	333	×10³/µL
Urea	0.29	g/L
Serum creatinine	7.00	mg/L
AST (SGOT)	14.70	U/L
ALT (SGPT)	12.50	U/L
Gamma-GT	16.90	U/L
Total bilirubin	5.00	mg/L
Indirect bilirubin	4.00	mg/L
Direct bilirubin	1.00	mg/L
Sodium (Na⁺)	138	mmol/L
Potassium (K⁺)	3.76	mmol/L

**Figure 2:**
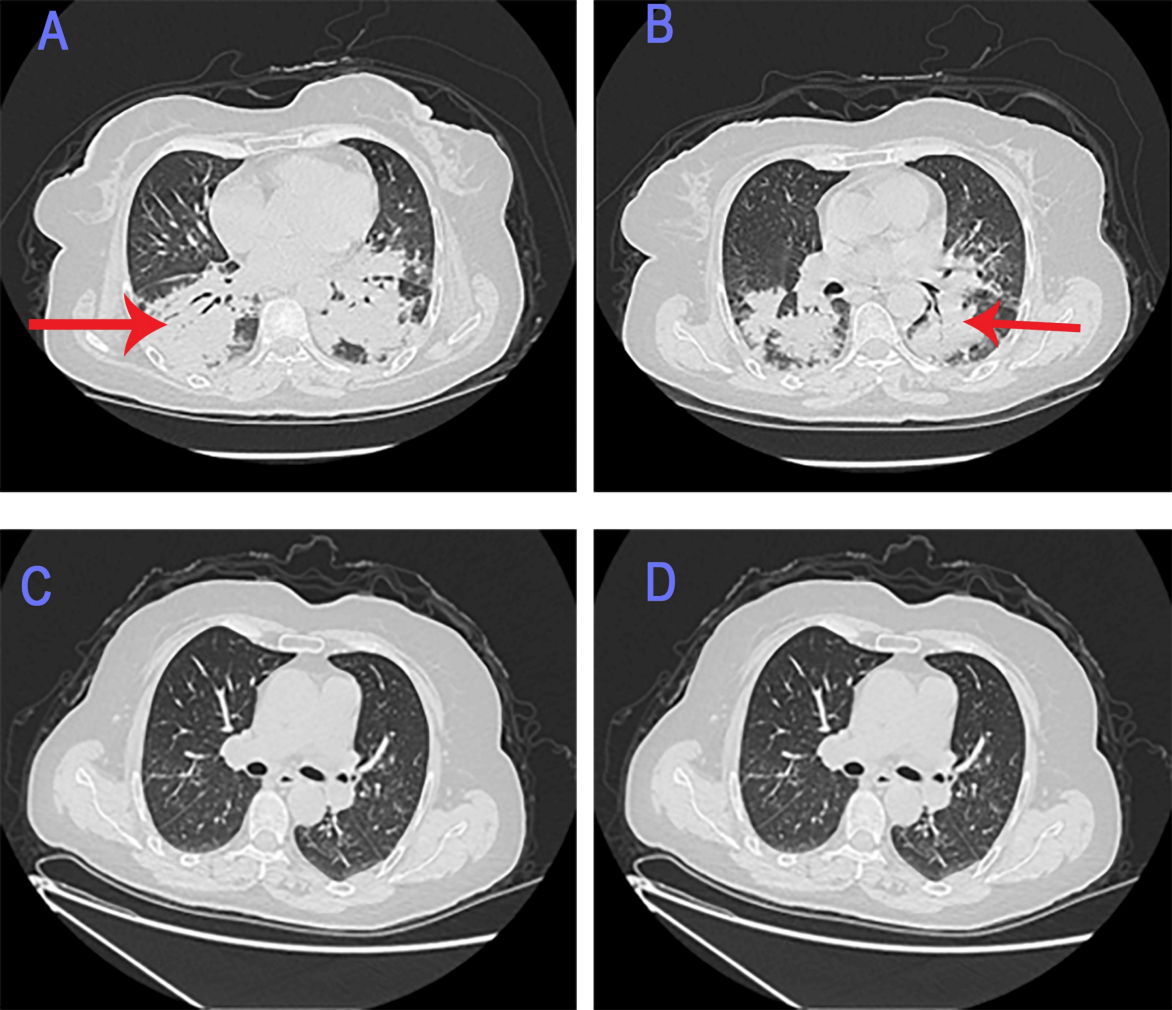
Chest CT scan. Chest CT scan showing bilateral alveolar syndrome (red arrow) before treatment (A and B) and resolution of the alveolar syndrome after treatment (C and D).

The thoracic CT angiography was performed due to elevated D-dimer levels at 5,700 mg/mL, showing bilateral pulmonary consolidation without pulmonary embolism (Figures 2A, 2B).

An electrocardiogram and a normal transthoracic echocardiogram ruled out cardiac decompensation, considering the acute clinical and radiological findings following sedation. Fluid overload was also excluded. The patient was started on oxygen therapy and received intravenous furosemide at a dose of 40 mg twice daily.

The clinical course showed rapid clinical improvement: no signs of respiratory distress. SpO₂: 94% on room air, respiratory rate: 24 breaths per minute; no crackles were heard on auscultation.

**Figure 3:**
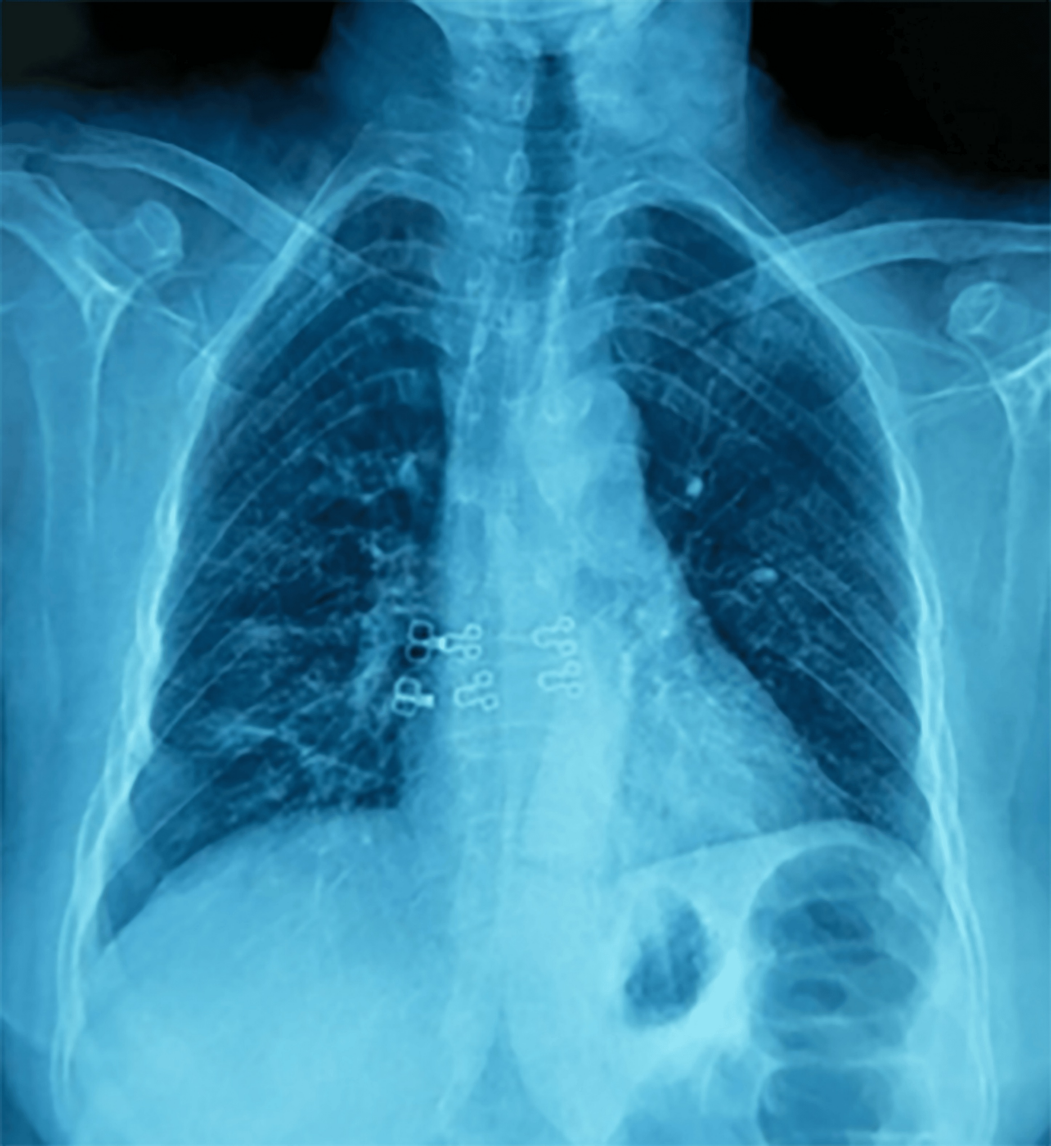
Radiological evolution after three days of treatment (frontal chest X-ray).

A second chest X-ray on the second day of treatment demonstrated good resolution of the initial alveolar syndrome (Figure 3).

The follow-up chest CT performed after one week also revealed radiological resolution of the alveolar syndrome (Figures 2C, 2D).

The sudden onset of clinical and radiological signs following upper GI endoscopy under sedation, in the absence of cardiac abnormalities on echocardiography, and rapid response to oxygen therapy and diuretic treatment administered over three days, supported the diagnosis of negative pressure pulmonary edema (NPPE) [1].

At the six-month follow-up after recovery, the patient remained in good health.

## Discussion

The first description of the pathophysiological relationship between the creation of negative pressure and the development of pulmonary edema was made in 1942 by Warren et al. [2,3]. The report by Oswalt et al. was the first to demonstrate the clinical significance of this phenomenon in three adult patients who developed pulmonary edema within minutes to hours after severe acute obstruction of the upper airways [4]. Since then, APENP has mostly been reported by anesthetists as a consequence of postoperative laryngospasm.

The diagnosis of NPPE requires an exclusion of alternative etiologies, such as A cardiac origin, which should be investigated first, especially in the presence of cardiovascular risk factors. Clinically, the patient presents with tachypnea, dyspnea, oxygen desaturation, rales, and/or clinical signs of left heart failure. The electrocardiogram findings, such as recent arrhythmias or ST-segment changes, further support this diagnosis of cardiogenic pulmonary edema.

The next step is to assess for fluid overload or inadvertent administration of hypotonic fluids. If this hypothesis is excluded, a recent neurological event such as traumatic brain injury, cerebral hemorrhage, or intracranial mass must be excluded. The presence of one of these factors suggests neurogenic acute hypertension as the underlying etiology. If a neurological origin has been ruled out, we must think about anaphylaxis, which must be considered through clinical evaluation for hypotension, skin rash, edema, bronchospasm, or exposure to an allergen typically associated with elevated serum tryptase levels. If anaphylaxis is excluded, the presence of acute airway obstruction should be assessed. When confirmed, the edema is most likely due to excessive negative intrathoracic pressure, indicating NPPE. Finally, if none of the above causes are identified, one should consider acute respiratory distress syndrome (ARDS).

Indeed, acute obstruction of the upper airways can lead to a series of complex consequences. Inspiratory forces can worsen this obstruction, generating significant intrathoracic negative pressure ranging from -50 to -100 cm H_2_O (normal is -3 to 10 cm H_2_O). This, in turn, increases venous return to the heart (preload) with a simultaneous decrease in cardiac output, associated with reduced pulmonary venous drainage to the left atrium. Pulmonary capillary pressures rise while intra-alveolar pressures decrease, disrupting alveolar cell junctions. The hydrostatic gradient between the interstitial space and the pulmonary capillary bed promotes fluid extravasation, contributing to the formation of pulmonary edema, which can persist even after relief of the airway obstruction [5]. When a critical amount of edema fluid accumulates in the interstitial compartment, alveolar flooding occurs [1].

Several factors are associated with postoperative sedation-induced laryngospasm, being the most frequent cause, followed by curarization and residual anesthesia [5,6].

Several studies have been published regarding this disease. One case involved a patient who developed APENP immediately after extubation following general anesthesia for an appendectomy [5]. Another report described a young patient who underwent total thyroidectomy for multinodular goiter, with the postoperative period marked by the onset of bilateral recurrent paralysis, which progressed to APENP [7]. Another case, reported in 2017, involved a 28-year-old woman who developed dyspnea followed by ARDS with unilateral pulmonary edema after extubation following general anesthesia for laparoscopic cholecystectomy [8].

The treatment of APENP remains a subject of debate; it often includes ventilatory support. In severe cases, intubation with positive pressure ventilation is required to improve gas exchange by alveolar recruitment. Non-invasive ventilation remains an alternative for moderate respiratory distress. Regarding medical treatment, diuretics are commonly used, although their use remains controversial. They are often administered in moderate doses to help reduce fluid accumulation in the lungs. It is important to note that each case is unique, and the treatment plan should be individualized [7,9,10]. The use of systemic corticosteroids and inhaled beta-2 agonists has also been reported [7,11].

In the first two studies, management required the use of non-invasive ventilation for favorable outcomes. In contrast, our case involved an older patient. "It is more common in young, healthy individuals for the development of strong negative intrathoracic pressures in response to upper airway obstruction" [10], and the case was related to sedation for a digestive endoscopy, which makes the case more unique. The patient had a favorable outcome with only medical treatment based on diuretics (Furosemide 80 mg injectable solution) and Salbutamol nebulization, which was also reported in the previous case [8].

## Conclusions

NPPE can be a severe complication that threatens vital prognosis, usually occurring after general anesthesia with orotracheal intubation, but may also develop following minor surgical procedures performed under sedation. A good understanding of this pathological entity allows for rapid diagnosis and appropriate management to avoid its potentially lethal consequences.
